# Health Related Quality of Life among TB Patients: Question Mark on Performance of TB DOTS in Pakistan

**DOI:** 10.1155/2018/2538532

**Published:** 2018-02-12

**Authors:** Madeeha Malik, Rida Nasir, Azhar Hussain

**Affiliations:** Hamdard Institute of Pharmaceutical Sciences, Hamdard University, Islamabad, Pakistan

## Abstract

Due to long duration of treatment and use of several agents, tuberculosis can lead to poor health related quality of life among patients.* Objective.* The present study was designed to assess health related quality of life among pulmonary tuberculosis patients in Pakistan.* Methodology.* A descriptive cross-sectional study design was used. SF-36 was self-administered to a sample of 382 pulmonary tuberculosis patients receiving self-administered or directly observed types of treatment, in baseline, initial, or continuous phase of treatment. After data collection, data was cleaned, coded, and statistically analyzed using SPSS version 21.0.* Results.* The results of the current study highlighted a significant impact on several domains of HRQoL of pulmonary TB patients. Highest HRQoL scores had been observed for the domain of physical functioning (60.03, ±25.779) whereas lowest HRQoL scores were observed for the domain of general health (34.97, ±14.286) perceptions of TB patients followed by bodily pain (43.40, ±24.594).* Conclusion.* The results of the present study concluded that TB patients had poor HRQoL in spite of the new therapeutic strategies and free availability of medicines. The disease had a negative impact on HRQoL of TB patients across all domains.

## 1. Introduction

Tuberculosis (TB) remains a major public health and economic problem worldwide [1]. World Health Organization declared TB alongside HIV as a leading cause of death killing almost 1.5 million people in the year 2014 and most of these deaths occurred in the developing world [2]. Increased knowledge and awareness about the disease is important along with early detection, diagnosis, and treatment in order to control TB. Though much attention has been given to clinical outcomes of therapy and microbiological cure, patient reported health related quality of life which can have a major influence on the clinical outcomes has been neglected. Treatment decisions should be based on patient preferences, which are also related to mental and social health in addition to physical health, to achieve better clinical outcomes [3]. The untreated patients can become a source of transmission of infection leading to spread of disease whereas irregularities in treatment can lead to drug resistance. Health related quality of life is the impact that perceived health status has on the normal functions of life. Reduced health related quality of life can lead to depression and medication nonadherence which can further lead to worsening of the medical condition. Tuberculosis has been one of the leading causes of mortality and morbidity in the world especially in the developing countries. Conventionally the effectiveness of tuberculosis treatment has always been observed and measured in terms of clinical outcomes. However, it may also lead to disease-related depression. So with the emergence of new strategies for treatment and control the focus of TB management has been shifted from mortality to disease-related morbidity and patient reported quality of life. Twenty-two countries carry 80% of the global incident cases of tuberculosis (TB). These countries were designated high-burden countries (HBCs) by the World Health Organization (WHO) in 1998, and they have received accelerated assistance to increase case detection rates and improve treatment outcomes. All of the HBCs had adopted WHO's DOTS strategy by 2000. By 2007, national tuberculosis programs (NTPs) in all HBCs had achieved 100% DOTS coverage nationwide, providing access to standardized TB care through public sector health facilities [4].

Tuberculosis is an infectious chronic disease that is a major public health problem globally. Pakistan ranks fifth among high-burden countries for tuberculosis. In 1994, the Ministry of Health of Pakistan in collaboration with WHO revised the TB control policy. National policy and technical guidelines were drafted. In 1995 the Ministry of Health decided on the location of 5 DOTS pilot sites in Pakistan, but only 1 site became operational. In 1996 the Directorate for TB Control of Pakistan was abolished and the MS of the TB Centre in Rawalpindi was made responsible for National TB Control program, without any additional support. In 1998 Pakistan was declared 1 of the 16 countries without an appropriate NTP. Recently it was decided that each province would be responsible for planning and managing its own NTP under Federal NTP guidelines. Funding for the plans will be provided through SAPP II comprehensive district healthcare system. Long duration of treatment period can have an effect on patient's life. Moreover, due to its contagious nature, tuberculosis is still associated with social stigma. Furthermore, social isolation associated with TB and burden of treatment subsequently have an effect on patient's health related quality of life. Very few studies have been carried out to focus on quality of life among tuberculosis patients. Very limited data is available regarding health related quality of life of tuberculosis patients in developing countries especially in Pakistan. Even after initiation of directly observed therapy (DOT) and free availability of medicines in Pakistan, control and eradication of TB could not be achieved; this raises concerns regarding performance of TB DOTS in Pakistan. Therefore, this study was designed to assess health related quality of life including physical health, general health perception, emotional health, psychological health, and social functioning of tuberculosis patients of Pakistan.

## 2. Methodology

A descriptive cross-sectional study design was used to assess HRQoL and its domains such as physical functioning, bodily pain, general health, role limitation, and mental health of pulmonary tuberculosis patients in Pakistan. Ethical approval was obtained from Ethical research Committee of Hamdard University. Moreover, in Pakistan, questionnaire-based studies do not need any endorsement from Ministry of Health. Despite that, prior information was sent to the Ministry of Health, Government of Pakistan, for the execution of this research. For data collection approval from MS of the hospitals was taken. Informed and verbal consent for participation were also taken from the respondents. Respondents were ensured about the confidentiality of information verbally as well as confidentiality undertaking being signed by the principal investigator. Study site for this research was public and private healthcare facilities treating TB located in Rawalpindi and Islamabad. The sampling frame comprised TB patients treated in public and private healthcare facilities in two cities of Pakistan. Study respondents included patients suffering from pulmonary tuberculosis. Patients suffering from pulmonary tuberculosis, aged 18 years or above, receiving self-administered or directly observed types of treatment, in baseline, initial, or continuous phase of treatment, smokers and nonsmokers, were included in this study. Patients aged less than 18 years, patients with extrapulmonary or miliary tuberculosis, and patients with any compelling conditions were excluded from this study.

### 2.1. Sample Size and Sampling Procedure

Calculation of sample size was performed by using Rao soft sample size calculator to determine the size of sample representing the population of pulmonary tuberculosis patients. Sample size was calculated as 382 to achieve 95% confidence interval with 5% margin of error. As no list of tuberculosis patients was available, convenience sampling technique was used to select the respondents. According to convenience sampling all the respondents that were available at time of data collection were selected.

### 2.2. Data Collection Tool

Prevalidated data collection tool Short form health survey (SF-36) was used. Written permission had been obtained from Optum. The tool was slightly modified according to study objectives and sociodemographics of our country. SF 36 included eight domains including physical functioning, role limitation due to physical problems, bodily pain, general health, vitality, social functioning, role limitation due to emotional problems, and emotional well-being. Respondent was greeted and evaluated. If the respondent did not read English or was bilingual, approved language version to use was determined or interviewer administration was used. If visual problems existed, a large-font form was administered or interviewer administration was used. The survey was introduced. Survey form was given to the respondent. Respondent was instructed on how to fill out the form. Any respondent questions were answered before, during, or after the administration. Form was retrieved upon completion and checked for completeness before the respondent left. Finally, respondent was thanked for completing the form.

### 2.3. Scoring of the Tool

Item response data was entered. Scoring of the SF-36v2 was begun with ensuring that the survey form was complete and the respondent's answers were unambiguous. The *n* item response values were recorded. Several steps were included in this process, including changing out of range values to missing, recoding values for 10 items, and substituting person-specific estimates for missing items. After item recoding, a total raw score was then computed for each health domain scale. The total raw score is the simple algebraic sum of the final response values for all the items in a given scale. Health domain scale total raw scores were transformed to 0–100 scores using the following formula: ((Actual raw score − Lowest possible raw score)/Possible raw score range) × 100. Health domain scale 0–100 scores were transformed to *T* scores using health domain *z* scores. A linear *z*-score transformation is used so that each health domain scale has a mean of 0 and a standard deviation of 1 with mean from the 0–100 score for that scale, and then dividing the difference by the given scale's standard deviation. Then *z* scores were transformed to *T* score. To do so, each *z* score was multiplied by 10, and then 50 was added to the resulting product. Health domain *z* scores were used to score Physical and Mental Component Summary measures.

### 2.4. Reliability and Validity of Tool

SF-36 is a prevalidated tool but still two focus group discussions had been conducted at different time intervals with experts from hospitals, academia, and regulatory and pharmaceutical industries for face and content validation of the tool. Beside this pilot testing had been conducted at 10% of the sample size to test the reliability of the tool after data collection. The value of Cronbach's alpha was 0.977 for SF-36, which was satisfactory considering that 0.68 is the cutoff value being disapproved.

### 2.5. Data Collection and Analysis

Data was collected by the principal investigator. The respondents were identified and after obtaining written/verbal consent from them, the questionnaire was hand delivered to them. The questionnaire was collected back on the same day to avoid study biasness. After data collection, data was cleaned, coded, and entered in SPSS version 21.0. Skewness test was performed and histograms with normal curves were used to check the normal distribution of data. Descriptive statistics comprising frequency and percentages was calculated. The nonparametric tests including Mann–Whitney and Kruskal–Wallis (*P* ≥ 0.05) were performed to find out the difference among different variables.

## 3. Results

### 3.1. Demographic Characteristics

Out of 382 respondents, 46.6% (178) were male and 53.4% (204) were female. Of the total respondents, 16.8% (64) were illiterate and 39.5% (151) were matric. Regarding the job status of the respondents, 38.2% (146) were employed whereas 30.6% (117) were unemployed. Out of all the respondents 22.5% (86) were smokers whereas 77.5% (296) were nonsmokers. Out of all the respondents, 86.1% (329) had duration of illness of less than 1 year. Of the total respondents 34.8% (133) were in initial phase of treatment. Regarding the type of treatment, 51.6% (197) respondents were in self-administered type of treatment and 48.4% (85) were under directly observed therapy. A detailed description of demographic characteristics is given (Table 1).

### 3.2. Domains of Health Related Quality of Life (HRQoL)

The results highlighted that lowest scores for HRQoL were observed in the domain of general health (34.97 ± 14.286) followed by domain of bodily pain (43.40 ± 24.594) whereas highest scores were observed in the domain of physical functioning (60.03 ± 25.779). A detailed description is given in (Table 2).

### 3.3. Comparison of HRQoL Domains by Demographic Characteristics

Comparison of HRQoL domains in different age groups demonstrated a significant difference (*P* ≥ 0.05) with TB patients aged more than 50 years having lower HRQoL scores. Analyzing the scores of the respondents with different education levels reported a significantly higher MCS (*P* ≥ 0.05) in TB patients having intermediate education. Significant difference (*P* ≥ 0.05) was found in PCS of TB patients having different job status with employed patients having better HRQoL. Furthermore, comparison of HRQoL domains across different phases of treatment revealed a significant difference (*P* ≥ 0.05) in HRQoL with poor HRQoL reported at baseline. A detailed description is given in (Table 3).

## 4. Discussion

Tuberculosis has remained a major public health problem worldwide resulting in increased morbidity and mortality. Due to prolonged therapy and infectious nature of the disease, physical, mental, and social distress are common among TB patients leading to poor disease outcomes. The results of the current study highlighted a significant impact on several domains of HRQoL of pulmonary TB patients. Highest HRQoL scores had been observed for the domain of physical functioning whereas lowest HRQoL scores were observed for the domain of general health perceptions of TB patients followed by bodily pain and role limitations due to physical problems. Majority of TB patients enrolled in the current study considered their health to be fair. These findings are in line with a study conducted in Sudan where two-thirds of TB patients reported their health between good and fair [5]. Similarly highest scores were observed in the physical functioning domain of SF-36 and lowest scores were observed in the general health perception area of WHOQOL-100 for TB patients in Turkey [6].

Constraints in physical functioning of TB patients are important to be considered on the account of their widespread prevalence that can lead to decreased quality of life and increased risk of depression and disability. The results of the present study revealed that vigorous activities such as running or lifting heavy objects, climbing several flights of stairs, and walking more than a km were limited a little for most of the TB patients. Most of the time they had to cut down time spent on work and accomplished less than they would have liked to achieve. Similar results were reported in a study conducted in Sudan where TB affected long distance movements of the TB patients and their activities were limited due to their health [5]. Vitality in TB patients reflects the feelings of having energy as compared to fatigue. Bodily pain can interfere with normal activities of TB patients and subsequently affect their quality of life. The results of the present study showed that most TB patients felt tired and did not have a lot of energy most of the time. Bodily pain had mild to moderate effect on the daily activities of most TB patients. Similar situation has been reported in Sudan where pain had moderate effect on patient's quality of life [5]. Tuberculosis can have an impact on social functioning due to social stigma associated with it. The results of the present study showed that social functioning of most of the TB patients was slightly affected. Furthermore, most of the TB patients had to cut down time for work, accomplished less, and did work and other activities less carefully only sometimes due to emotional problems. Similar findings were reported from a study conducted in Sudan where TB patients felt that their health had little effect on their social relations [5].

The results of the present study highlighted that patients aged more than 50 years had impaired HRQoL across all domains. This might be due to the fact that health declines with aging process. These results are in line with study conducted in China that also showed association between age and HRQoL [7]. Along the same lines, studies conducted in Malaysia and Canada also reported lower physical health in elderly patients [8, 9]. The results of the present study indicated that females had poor physical health as compared to males. This might be due to the fact that women are more sensitive to changes in their health and have low levels of physical strength. Similar results were reported in a study conducted in USA where women had more health problems and were more likely to report fair or poor health than men [10]. The results of the present study revealed that QoL scores for unmarried patients were lower than those of married patients; however, the results were not significant. Similarly, married subjects in Turkey had higher QoL scores as compared to unmarried subjects but the results were not significant [11]. The results of the present study highlighted that TB patients with intermediate or higher education had better mental component summary score as compared to illiterate patients. The results of the present study showed that socioeconomic status has an impact on HRQoL of TB patients. Patients earning between Rs. 21 and 35,000 per month had better physical health whereas patients earning between Rs. 10 and 20,000 had better mental health. Unemployed TB patients had poor physical HRQoL as compared to employed TB patients. This might be due to the fact that education leads to more adaptability in life and motivation for self-care which overall improves vitality and social functioning. Moreover, high literacy can lead to improved employment status and financial and social matters resulting in better health seeking behavior. Studies conducted in Turkey, South Africa, Thailand, and Iraq also reported that higher QoL was associated with higher education level and better income [6, 11–14]. On the other hand, it was interesting to notice no significant difference among TB smokers and nonsmokers enrolled in current study. However, these findings are in contrast to other studies conducted in Iran and Thailand in which cigarette smoking was associated with lower HRQoL especially in the domain of social functioning [1, 14].

The results of the present study revealed that TB patients suffering from disease for more than two years had poor physical health. TB patients at baseline had lowest HRQoL followed by patients undergoing initial phase of treatment. Furthermore, TB patients taking treatment for less than 1 month reported poor HRQoL as compared to TB patients taking treatments for longer periods of time. TB patients taking treatment for 4–6 months had better mental health whereas TB patients taking treatment for 7–9 months had better physical health. This might be due to the reduction in TB symptoms and positive effect of therapeutic interventions on HRQoL of TB patients. These findings are in accordance with studies conducted in Canada and Iran that also reported poor HRQoL at baseline with improvement after 2 months of therapy [15–17]. The current study also reported that patients undergoing DOTS did not show any significant difference in physical functioning, role limitations due to physical problems, and general health perception as compared to patients undergoing self-administered type of therapy. However, treatment plans incorporating directly observed therapy from the start had showed better treatment outcomes in different countries [18].

## 5. Limitations of the Study

This study was conducted in the two cities of Pakistan and results may not be generalizable to other parts of the country. Time and financial constraints were also faced during the conduction of study.

## 6. Conclusion

The results of the present study concluded that TB patients had poor HRQoL in spite of the new therapeutic strategies and free availability of medicines. The disease had a negative impact on HRQoL of TB patients across all domains. HRQoL of females and patients aged more than 50 years was found to be more affected due to TB. Beside this less educational qualification and duration of disease had negative effect on mental HRQoL of TB patients while better socioeconomic status showed positive effect on HRQoL of TB patients. All the stakeholders need to work together for improving physical and mental health related quality of life of TB patients in order to improve medication adherence, well-being, and functioning of TB patients. The poor health related quality of life among TB patients in Pakistan raises serious concerns on performance of TB DOTS program and calls for revamping the program in terms of its effectiveness.

## 7. Future Implications

Extensive research should be conducted to design appropriate interventions for improving HRQoL in TB patients to decrease the rates of treatment failures and improve treatment response. Interventional studies focusing on health educational programs targeting patients with low educational background must be designed. Studies assessing HRQoL of TB patients at baseline and initial phase of treatment to prevent treatment defaults must be conducted.

## Figures and Tables

**Table 1:** Demographic characteristics.

Indicator	Total % (*n*)
Age	
18–30 Y	53.9 (206)
31–40 Y	31.2 (119)
41–50 Y	10.2 (39)
>50 Y	4.2 (16)
Gender	
Male	46.6 (178)
Female	53.4 (204)
Marital status	
Married	58.6 (224)
Unmarried	36.9 (141)
Divorced	2.6 (10)
Widowed	1.8 (7)
Qualification	
Illiterate	16.8 (64)
Primary	33.8 (129)
Matric	39.5 (151)
Intermediate	9.7 (37)
Bachelors	0.3 (1)
Masters	0 (0)
Ph.D.	0 (0)
Job status	
Employed	38.2 (146)
Unemployed	30.6 (117)
House keeper	29.1 (111)
Retired	2.1 (8)
Cigarette smoking	
Smoker	22.5 (86)
Nonsmoker	77.5 (296)
Current salary	
Rs. < 10,000	24.1 (92)
Rs. 10,000–20,000	56 (214)
Rs. 21,000–35,000	16.8 (64)
Rs. 36,000–50,000	3.1 (12)
Rs > 50,000	0 (0)
Duration of illness	
<1 Y	86.1 (329)
1.1–2 Y	12 (46)
2.1–3 Y	0.8 (3)
3.1–5 Y	0 (0)
5.1–10	0 (0)
>10 Y	1 (4)
Duration of treatment	
<1 month	29.1 (111)
1–3 months	34 (130)
4–6 months	27.2 (104)
7–9 months	4.7 (18)
10–12 moths	3.4 (13)
>13 months	1.6 (6)
Phase of treatment	
Baseline	28.5 (109)
Initial phase	34.8 (133)
Continuous phase	36.6 (140)
Type of treatment	
Self-administered	51.6 (197)
Directly observed therapy	48.4 (185)

**Table 2:** Mean scores for different domains of health related quality of life (HRQoL).

Indicator	Mean	Median	Standard deviation
Physical functioning	60.03	65.00	25.779
Role physical	45.37	37.50	25.726
Bodily pain	43.40	44.44	24.594
General health	34.97	33.33	14.286
Social functioning	58.64	62.50	26.509
Role emotional	59.01	58.33	26.285
Vitality	57.38	56.25	15.398
Mental health	57.89	55.00	16.552

**Table 3:** Comparison of HRQoL domains by demographic characteristics.

Demographics	Physical health component score				Mental health component score				Composite score			
	*n*	Mean rank	Test statistics	*P* value	*n*	Mean rank	Test statistics	*P* value	*n*	Mean rank	Test statistics	*P* value
Gender	Male = 178	203.12	16087.00^a^	0.055	Male = 178	200.31	16588.000^a^	0.145	Male = 178	202.06	16276.500^a^	0.081
	Female = 204	181.36			Female = 204	183.81			Female = 204	182.29		

Marital status	Married = 224	184.64	15424.50^a^	0.708	Married = 224	187.46	14793.000^a^	0.309	Married = 224	185.59	15211.500^a^	0.554
	Unmarried = 141	180.39			Unmarried = 141	175.91			Unmarried = 141	178.88		

Age	18–30 yrs = 206	197.57	19.384^b^	0.001	18–30 yrs = 206	193.63	16.187^b^	0.003	18–30 yrs = 206	196.03	18.049^b^	0.001
	31–40 yrs = 119	191.49			31–40 yrs = 119	189.54			31–40 yrs = 119	189.93		
	41–50 yrs = 39	199.97			41–50 yrs = 39	214.88			41–50 yrs = 39	208.09		
	More than 50 yrs = 16	79.12			More than 50 yrs = 16	102.41			more than 50 yrs = 16	88.12		

Qualification	Illiterate = 64	161.10	7.523^b^	0.111	Illiterate = 64	150.87	10.857^b^	0.028	Illiterate = 64	154.77	9.412^b^	0.052
	Primary = 129	192.40			Primary = 129	200.18			Primary = 129	195.74		
	Matric = 151	197.98			Matric = 151	196.83			Matric = 151	197.78		
	Intermediate = 37	216.14			Intermediate = 37	208.64			Intermediate = 37	214.78		

Job status	Employed = 146	210.33	11.408^b^	0.010	Employed = 146	208.06	7.235^b^	0.065	Employed = 146	209.50	9.317^b^	0.025
	Unemployed = 117	185.68			Unemployed = 117	183.38			Unemployed = 117	184.53		
	House keeper = 111	179.48			House keeper = 111	182.85			House keeper = 111	180.74		
	Retired = 8	99.75			Retired = 8	128.12			Retired = 8	114.25		

Cigarette smoking	Smoker = 86	196.30	12315.50^a^	0.647	Smoker = 86	198.03	12166.000^a^	0.533	Smoker = 86	195.16	12413.000^a^	0.727
	Nonsmoker = 296	190.11			Nonsmoker = 296	189.60			Nonsmoker = 296	190.44		

Current salary	Rs. < 10,000 = 92	154.32	21.683^b^	0.000	Rs. < 10,000 = 92	162.47	14.369^b^	0.002	Rs. < 10,000 = 92	156.36	18.205^b^	0.000
	Rs. 10–20,000 = 214	203.99			Rs. 10–20,000 = 214	208.01			Rs. 10–20,000 = 214	207.04		
	Rs. 21–35,000 = 64	216.77			Rs. 21–35,000 = 64	188.66			Rs. 21–35,000 = 64	202.10		
	Rs. 36–50,000 = 12	119.08			Rs. 36–50,000 = 12	134.67			Rs. 36–50,000 = 12	127.29		

Duration of illness	<1 Y = 329	191.27	9.744^b^	0.021	<1 Y = 329	191.23	3.765^b^	0.288	<1 Y = 329	191.18	6.850^b^	0.077
	1.1–2 Y = 46	205.03			1.1–2 Y = 46	198.62			1.1–2 Y = 46	202.37		
	2.1–3 Y = 3	226.83			2.1–3 Y = 3	239.67			2.1–3 Y = 3	238.00		
	>10 Y = 4	28.25			>10 Y = 4	95.88			>10 Y = 4	57.75		

Duration of treatment	<1 month = 111	99.08	145.056^b^	0.000	<1 month = 111	111.42	139.812^b^	0.000	<1 month = 111	100.40	152.007^b^	0.000
	1–3 months = 130	189.25			1–3 months = 130	171.53			1–3 months = 130	182.19		
	4–6 months = 104	260.94			4–6 months = 104	274.74			4–6 months = 104	269.16		
	7–9 months = 18	298.25			7–9 months = 18	272.75			7–9 months = 18	288.33		
	10–12 months = 13	280.04			10–12 months = 13	272.04			10–12 months = 13	282.42		
	>13 months = 6	234.25			>13 months = 6	244.50			>13 months = 6	245.00		

Phase of treatment	Baseline = 109	97.85	137.859^b^	0.000	Baseline = 109	109.56	135.088^b^	0.000	Baseline = 109	98.75	146.589^b^	0.000
	Initial phase = 133	192.53			Initial phase = 133	175.21			Initial phase = 133	185.73		
	Continuous phase = 140	263.44			Continuous phase = 140	270.76			Continuous phase = 140	269.19		

Type of treatment	Self-administered = 197	182.46	16441.500^a^	0.099	Self-administered = 197	192.17	18090.000^a^	0.902	Self-administered = 197	187.50	17435.000^a^	0.465
	DOT = 185	201.13			DOT = 185	190.78			DOT = 185	195.76		

^a^Mann–Whitney test; ^b^Kruskal–Wallis test (*P* ≥ 0.05).
